# A Review of Temperature, pH, and Other Factors that Influence the Survival of *Salmonella* in Mayonnaise and Other Raw Egg Products

**DOI:** 10.3390/pathogens5040063

**Published:** 2016-11-18

**Authors:** Thilini Piushani Keerthirathne, Kirstin Ross, Howard Fallowfield, Harriet Whiley

**Affiliations:** School of the Environment, Health and the Environment, Flinders University, GPO BOX 2100, Adelaide 5001, Australia; keer0004@flinders.edu.au (T.P.K.); kirstin.ross@flinders.edu.au (K.R.); howard.fallowfield@flinders.edu.au (H.F.)

**Keywords:** *Salmonella* spp., salmonellosis, mayonnaise, raw egg products, NaCl, pH, garlic, oils, fat content

## Abstract

Salmonellosis is one of the main causes of foodborne illnesses worldwide, with outbreaks predominately linked to contamination of eggs and raw egg products, such as mayonnaise. This review explores previous studies that have investigated *Salmonella* control mechanisms utilized in the production of raw egg mayonnaise and other food products. Apart from the use of pasteurized eggs, the main control mechanism identified is the pH of the raw egg products, which plays an important role in the consistency and stability while affecting the survival of *Salmonella* spp. However, currently there is no consensus regarding the critical pH limit for the control of *Salmonella.* The effectiveness of pH as a control mechanism is influenced by the type of acid used, with the effectiveness of lemon juice compared with vinegar highly debated. Additionally, *Salmonella* susceptibility to pH stresses may also be influenced by storage temperature (in some studies refrigeration temperatures protected *Salmonella* spp. from acidulants) and is further complicated by the development of *Salmonella* cross-tolerance-induced responses, pH homeostasis achieved by the cellular antiport and symport systems, and acid tolerance response (ATR). These mechanisms all provide *Salmonella* with an added advantage to ensure survival under various pH conditions. Other confounding factors include the fat content, and the addition of NaCl, garlic and plant essential oils (PEOs) from mint, cinnamon, cardamom and clove.

## 1. Introduction

Worldwide, foodborne illness is one of the most serious health problems affecting public health and development [1]. In the USA, it was estimated that the annual incidence of foodborne illness was 48 million cases and the economic burden estimate ranges from US $51.0–77.7 billion [2]. In Canada, annual estimates of foodborne illness range from 3.1–5.0 million cases [3] and in Australia the incidence is estimated at 5.4 million cases costing AUD$1.2 billion annually [4]. Worldwide, it is estimated that *Salmonella* is responsible for 80.3 million cases of food borne illness [5].

*Salmonella* spp. are members of the family Enterobacteriaceae [6] and are facultative anaerobic rods [7]. There are more than 2600 known serovars [8], and within a serovar the strains could be different in virulence [9]. The serovars of *Salmonella* are broadly classified into typhoidal and nontyphoidal Salmonella (NTS). Nontyphoidal *Salmonella* (NTS) serovars include Typhimurium and Enteritidis, which are pathogens with a wide-range of host specificity, but *S*. *enterica* serovars, Typhi, Sendai, and Paratyphi A, B, and C are highly adapted to the human as a host and are the causative agents of enteric fever [8]. While there are over 2600 identified serotypes of *Salmonella*, the majority of clinical *Salmonella* cases can be attributed to 20 serotypes [10], but there are instances where infection occurs due to uncommon serotypes of *Salmonella* [11].

One of the main sources of foodborne salmonellosis is eggs and raw egg products [12]. Outbreaks of *Salmonella* serotype Enteritidis have been repeatedly associated with the consumption of raw and undercooked eggs [13,14]. The eggs can be contaminated during different stages of their formation, processing, and packaging. Vertical or transovarian contamination of eggs can happen during the formation of the egg when the ovaries of the hen are infected. Horizontal transmission can take place if the eggs are contaminated by means of a contaminated environment [15]. According to previous studies, there is an increased probability of whole eggs with low shell quality being able to be penetrated by *Salmonella* spp. [16], and egg weight and flock age also influence the ability of *Salmonella* spp. to penetrate the shell and the membranes of the eggs [17]. Moreover, it is found that there is an increased incidence of eggshell penetration from the beginning to the end of lay by *S.* Enteritidis [18], and there is an increased risk of *Salmonella* contamination when the flock becomes older [19].

Even though several food items like peanuts, beef, pork, and chicken have been connected with the outbreaks of salmonellosis, eggs and food products prepared using eggs seem to be the most frequent foods that are involved with the disease [20]. Raw eggs are used in many food products like pastries, homemade ice cream, and mayonnaise. Contaminated eggs that have not undergone heat treatment pose a significant risk to public health, and outbreaks of *S.* Enteritidis have been repeatedly associated with raw or undercooked eggs [21].

In 2008, the OzFoodNet conveyed that 5.4 million cases of foodborne disease, occur in Australia annually, costing an estimated amount of $1.2 billion dollars per year, among which 8310 cases were found to be *Salmonella* infections at a rate of 39 cases per 100,000 population [4]. While improved sanitation and water supplies have made a significant contribution towards the decrease in incidence of enteric diseases, *Salmonella* spp. and *Campylobacter* spp. are commonly reported pathogens of community gastroenteritis [22].

Within raw egg products, homemade mayonnaise prepared with raw eggs is one of the most common foods linked to salmonellosis outbreaks [23]. Mayonnaise is a methodically prepared, semisolid (stabilized oil–water emulsion) dressing, which is a combination of raw eggs, vinegar, oil, and spices, and is perhaps one of the oldest and most widely used in the world [24,25]. Currently, in many countries it is not feasible to produce eggs guaranteed to be free from *Salmonella* contamination, and hence the control mechanisms utilized post-collections and during food handling are essential for protecting public health [12].

In the USA, it is estimated that there are around 1.4 million illnesses and 600 deaths caused by salmonellosis annually, with the most common serotypes identified as *S. typhimurium* and *S.* Enteritidis [26]. In fact, from 1998 to 2008 there were a total of 403 outbreaks of foodborne salmonellosis, and 112 were linked to eggs and raw egg products [27]. In 2009, it was estimated that there were a total of 6.2 million cases of salmonellosis in the European Union, and the incidence rate correlated with the prevalence of *S.* Enteritidis in laying hens [28]. In Australia in 2011, 45% of all salmonellosis outbreaks were linked to eggs or egg-related products [29]. Globally, there have been numerous published outbreaks of salmonellosis that have been caused by raw-egg mayonnaise [30]. In the U.K., a large outbreak of *Salmonella typhimurium* definitive type 49 was linked to eggs with raw-egg mayonnaise identified as the likely cause of infection [30]. Another outbreak of salmonellosis was also reported form the U.K. in December 2000, which identified egg mayonnaise sandwiches as the vehicle of infection [31]. Moreover, in Brazil, potato salad made with homemade mayonnaise and stored at unsuitable temperatures was associated with foodborne infection, and *S.* Enteritidis was identified as the infecting organism [32]. Additionally, in 2010, buffet dishes, which contained mayonnaise, was associated with a salmonellosis outbreak in Germany [33].

This review explores studies investigating different mechanisms for controlling *Salmonella* in raw-egg mayonnaise. The *Salmonella* serovar, country of study, and control mechanism investigated are presented in Table A1.

## 2. pH/Acid Tolerance and Temperature

**Figure A1:**
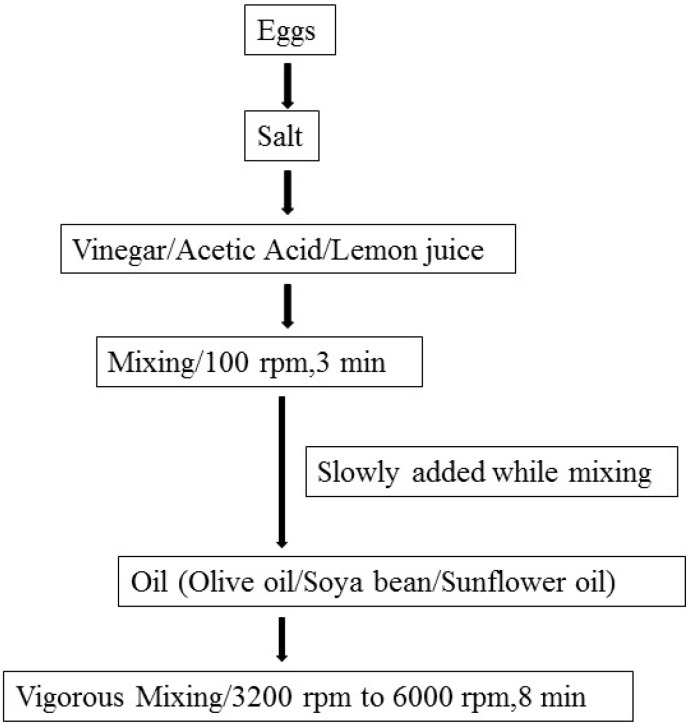
Preparation of mayonnaise [74,81,82,84,85].

The pH of mayonnaise plays an important role in its structure and stability [24]. Mayonnaise is an emulsion (Figure A1) stabilized by denatured proteins forming a network that can be impacted by the isoelectric pH of the egg yolk protein. When the charge on the proteins is minimized, the viscoelasticity and stability of the mayonnaise is at its highest [24].

Food safety guidelines published online by the Government of New South Wales (NSW), Australia, suggest that a pH at or below 4.2 has shown to be effective in controlling *Salmonella* in raw-egg products, however, there are numerous factors that influence the bactericidal efficiency such as the type of acid used [34,35], temperature [36], water activity [37], garlic, ginger, and pepper [38].

Many bacterial species induce responses to environmental stress [39]. When *Salmonella* spp. are exposed to a stress this can produce cross-tolerance to many or various stresses [40]. Gruzdev et al. reported that following carbon starvation, *Salmonella* spp. demonstrated greater tolerance to low pH, hyperosmolarity, heat, polymyxin B, and peroxides [41]. Another study conducted by Leyer and Johnson [42] demonstrated that exposure of *Salmonella* to mild acids (pH 5.8) could induce adaptation to lower pH, heat, NaCl (2.5 M), crystal violet, and polymyxin B. Additionally, subjecting *S. enterica* cells to an initial acid shock or pH 5.8 or 4.5 before inoculating mayonnaise (pH 4.2–4.5) increased the survival rate and persistence of the organism at 4 °C [43].

*Salmonella* can also achieve pH homeostasis, which is when the intracellular pH is maintained compared with the environmental pH [44]. Homeostasis is facilitated by cellular proton pumps and potassium/proton and sodium/proton antiport systems [45]. The ability of *Salmonella* to decrease proton extrusion and membrane proton conductance enables the cell to be protected against acid stress [46]. Additionally, *S. typhimurium* has a regulated response to further protect from acid stress, which is called the acid tolerance response (ATR) [47]. The ATR protects *Salmonella* spp. at pH levels of 3.0–4.0, but is activated when environmental pH values are between 6.0 and 5.5 and when pH homeostasis fails [47]. These pH conditions are referred to as the postshock stage and the preshock stage, respectively [47]. During the postshock stage, stimulation of 43 acid shock proteins takes place in order to prevent and repair the damage done to macromolecules by the acids [46].

In contrast, studies conducted by Álvarez-Ordóñez et al. [48] and Samelis et al. [49] suggest that *S. typhimurium* vulnerability to acid stress is dependent on growth temperature. *S*. *typhimurium* growth was observed in the temperature range of 25–37 °C at pH 4.5 [48,49]. Alali et al. [50] proposed that lowering the pH of the mayonnaise-based homemade salads decreased the rate of survival of *Salmonella* regardless of the temperature. According to a study conducted by Koutsoumanis et al. [51], the minimum pH value that permitted the growth of *S. typhimurium* was 3.94 within the temperature range 25–35 °C.

## 3. Vinegar vs. Lemon Juice

Jung and Beuchat [34] found that citric acid (lemon juice) was more effective at controlling *S. typhimurium* compared with acetic acid (vinegar/8.3 M), lactic acid (2 M), and malic acid (2 M) at an equivalent acid concentration of pH 5.4, 4.4, and 3.7. This is supported by work done by Zhu et al. (2012) which demonstrated that lemon juice was more effective than commercial wine vinegar at controlling *Salmonella* in mayonnaise spiked with either a mixture of *S.* Enteritidis (phage 4, 8, and 13) or a mixture of *S. typhimurium*, *S. heidelberg* and *S.* Enteritidis (untypeable phage type). It was also shown that both mixtures of *Salmonella* survived longer at 4 °C compared to 25 °C. The different bactericidal effects observed at different temperatures could be explained by more efficient cross-membrane migration of the organic acids at higher temperatures [36].

However, these findings differ from a study done by Perales and Garcia [52] that demonstrated that homemade mayonnaise made with vinegar had a greater bactericidal effect on *S.* Enteritidis compared with homemade mayonnaise made using lemon juice at the same pH (pH ranging from 3.6 to 5 [52]). This study was supported by Lock and Board [53], who also demonstrated that the number of *S.* Enteritidis PT4 organisms spiked into mayonnaise made with vinegar declined within six days of storage at 20 °C, but the same result was not observed when the mayonnaise was made to the same pH using lemon juice. Roller et al. (2000) concluded that chitosan added to mayonnaise containing acetic acid or lemon juice could be used as a preservative against the normal flora [54].

## 4. Addition of NaCl and Reduction of Water Activity

Several cellular mechanisms of bacterial cells are involved in osmoregulation, which regulates the osmolality of the cell, protecting physical and chemical properties of the intracellular environment in response to environmental stress [36]. It is achieved by accumulation of electrically neutral, low molecular weight compounds such as osmoprotectants (e.g., proline, glycine-betaine, or ectoine) inside the cell [55]. It is clear that *Salmonella* is adapted to endure prolonged starvation and desiccation periods [56]. It has been demonstrated that during the early stages of starvation, *Salmonella* can upregulate the osmoprotectant transporters (proP, proU, and osmU), ensuring the survival of this bacteria under low and intermediate moisture conditions [57].

Salt is a common preservative used in food products [58]. The sodium ions associate with water molecules to reduce the amount of unbound water in foods, making it difficult for the microorganisms to grow [59]. Salt can stimulate osmotic shock in microbial cells, affecting the growth and promoting cell death [60].

**Figure A2:**
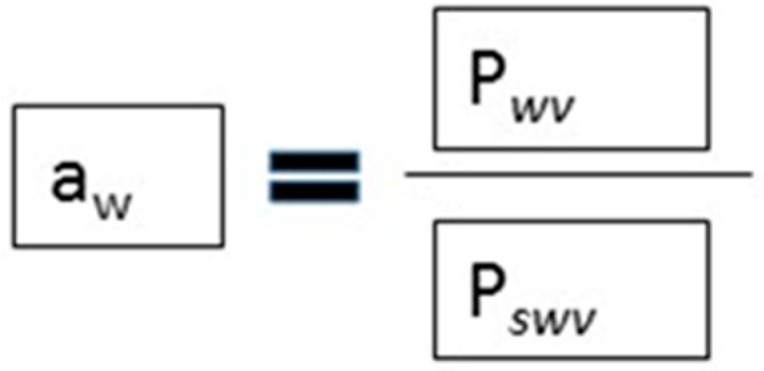
Definition of water activity.

It has been shown that the permeability of the *S*. *typhimurium* cells is altered by heat, and this allows the sodium ions to penetrate the cell into the cytoplasm and interfere with the cell metabolism [61]. Although there are limited studies investigating the effect of salt concentration on *Salmonella* contamination of mayonnaise, there have been studies demonstrating that *Salmonella* spp. are capable of enduring extended starvation and desiccation stresses [62]. Even though reducing the amount of available water in food is a long-established method for controlling bacterial growth [37], there have been outbreaks of salmonellosis linked to foods with low water activity (a*_w_*), such as peanut butter [56]. Water activity is defined as the ratio of water vapor pressure (P*_wv_*) in a food system to the saturation water vapor pressure (P*_swv_*) at the temperature of the food system (Figure A2). Optimal growth of *Salmonella* spp. occurs when the a*_w_* is 0.99, but there is evidence that *Salmonella* may develop increased tolerance that allows for survival under low a*_w_* conditions for longer periods time (i.e., 43 days) [37]. According to Mattick et al. [37], *Salmonella* spp. are found to have an increased heat tolerance at low a*_w_*.

## 5. Garlic (Allium sativum)

According to the scripts found on ancient Egyptian pyramids, garlic has been used for medicinal purposes as well as a spice since ancient times [63]. In 1822, the antibacterial properties of onion and garlic were observed and recorded [64]. According to the literature, garlic contains strong antibacterial compounds that are effective against *Salmonella* and is often used as a spice for fermented fish [65]. Use of garlic in food products could increase the shelf life as well as decrease the potential for food poisoning [63]. Garlic, ginger, and pepper are known to contain bactericidal agents against *Salmonella* [38]. Traditional medicine uses the extract of these plants in order to treat infections caused by enteric bacteria like *S. typhi* [66]. It has been demonstrated that addition of 1% garlic to the mayonnaise inoculated with acid tolerance-induced *S.* Enteritidis reduced counts of the organism by 10-fold after 2 days compared with the control mayonnaise batch incubated without garlic [67].

## 6. Oils

Plant essential oils (PEOs) are well-known as antibacterial agents that could be used to control foodborne diseases [68]. These plant secondary metabolites are hydrophobic in nature and can be added to mayonnaise, which is advantageous because they can interact with the cell membranes of the bacteria, subsequently causing the cell components to flow out from the cell [69]. According to Wendakoon et al. [70], some PEOs can hinder the enzymatic reactions through the inhibition of the proteins of bacteria [70]. PEOs from mint, cinnamon, cardamom, and clove were found to reduce the bacterial count of *S.* Enteritidis in milk products, yogurt, and cucumber [71]. In a study conducted by Dabbah et al. [72], PEOs from orange, lemon, and grapefruit reduced the *Salmonella* count in milk. According to Valverde et al. [73], cinnamon bark oil at the concentration of 7000 ppm can be used to decrease *Salmonella* spp. in liquid whole eggs. Additionally, it has also been found that the antimicrobial activity of oregano essential oils (OEO) is improved when combined with ethylenediaminetetraacetic acid (EDTA) or nisin [74]. The inhibitory activity of nisin can be increased when combined with EDTA, which alters the bacterial outer membrane, enabling nisin to access the cytoplasmic membrane [75].

## 7. Fat Content

Juneja and Eblen (2000) reported that increased fat content in food decreased the water activity, which could lead to poor heat conductivity, increasing the survival rate of the pathogen *S*. *typhimurium* in beef. This is supported by a study that showed the fastest decrease in *S. typhimurium* was detected in fat-free mayonnaise (at pH 2.6) compared with full-fat mayonnaise at the same pH [76].

## 8. Conclusions

*Salmonella* contamination of raw egg products, such as mayonnaise, is a major issue of public health concern as a causative agent of salmonellosis outbreaks. This review paper explores studies that have that have investigated *Salmonella* control mechanisms within raw-egg mayonnaise. One of the main factors influencing the survival of *Salmonella* in mayonnaise, whilst also affecting the appearance and stability, is pH. Some studies indicate that *Salmonella* susceptibility to pH stresses may be dependent on the growth temperatures; however, this is still debated. Additionally, the effectiveness of pH as a control mechanism is influenced by the type of acid used. The effectiveness of lemon juice compared with vinegar as a control mechanism is also still debated, with current studies producing contradicting results. Additionally, evaluating the effectiveness of pH as a control mechanism is further complicated by the development of *Salmonella* cross-tolerance-induced responses, pH homeostasis achieved by the cellular antiport and symport systems, and ATR, which provides *Salmonella* with an added advantage to ensure survival under various pH environments. There have also been a few studies investigating the effectiveness of additive such as salt, garlic, and PEOs from mint, cinnamon, cardamom, and clove to inhibit the growth of *Salmonella* spp.

Currently, it is not possible to guarantee that raw-egg mayonnaise will not be contaminated with *Salmonella.* Therefore, there is an urgent need for further research to continue to explore the potential control mechanisms discussed in this paper. This will inform protocols for food handling and mayonnaise preparation to reduce the risk of foodborne salmonellosis.

## Appendix A

**Table A1:** *Salmonella* serovar, country of study, and control mechanism investigated.

Country	*Salmonella* spp.	Food	Control Mechanisms	Comments	Reference
U.K.	*S*. Enteritidis	Mayonnaise	pH	20 mL vinegar (6% *w*/*v* acetic acid) per fresh egg yolk, 40 mL per fresh egg white or 60 mL per fresh whole egg was used. Should be held at 20 °C or above for at least 48 h before refrigeration or consumption.	[77]
U.K.	*S.* Enteritidis	Mayonnaise-based shrimp salad	pH/preservatives chitosan and acetic acid	Chitosan could be useful as a preservative combined with acetic acid.	[54]
China/U.S.	*S. typhimurium S. heidelberg S*. Enteritidis	Home-Style Mayonnaise	pH commercial wine vinegar, lemon juice, and acetic or citric acid	*Salmonella* counts in acid solutions at 4 °C were reported as significantly higher than those in samples at 25 °C. Viability of *Salmonella* decreased as the amounts of vinegar and lemon juice in mayonnaise increased.	[35]
France	*S*. *typhimurium*	Reduced-calorie mayonnaise	pH/Temperature	Higher temperature with a low pH, greater the inactivation of the organism.	[78]
U.S.	*Salmonella*	Mayonnaise-based potato salad, macaroni salad, and coleslaw	pH, NaCl, and temperature	Decreased pH and increased the bactericidal activity irrespective of sodium concentration or storage temperature Sodium concentrations had little or no effect on the behavior of *Salmonella* when stored at 4 or 10 °C for up to 27 days.	[50]
U.K.	*S.* Enteritidis	Mayonnaise	pH acetic acid (vinegar)	Mayonnaise made with vinegar to a pH of 4.1 or less controlled *S.* Enteritidis. Storage of mayonnaise at refrigeration temperatures protected *Salmonella* spp. from acidulants and therefore a holding time of 24 h at 18–22 °C was advised before refrigeration.	[79]
Spain	*S*. Enteritidis	Home-made Mayonnaise	pH/temperature	Vinegar was used as an acidulant to achieve a pH of 3.6–4 and storage in a warm place is recommended.	[52]
U.S.	*S*. *senftenberg*	Egg salads	pH/temperature	Significant decrease in *Salmonella* numbers, particularly during storage at room temperature (22 °C) at the acidity ranging from pH 4.25–4.30 was recorded.	[80]
Brazil	*S*. Enteritidis	Mayonnaise	oil oregano essential oil	Natural antimicrobial to reduce the *S*. Enteritidis growth	[81]
Spain	*S*. Enteritidis	Egg mayonnaise	oil (virgin olive oil)	Egg mayonnaise made with virgin olive oil required more than 48 h to reduce the number of microorganisms to an undetectable level.	[54]
U.K.	*S*. Enteritidis	Homemade Mayonnaise	Temperature, pH, oils (olive oil with garlic, basil, soya, grapeseed, rapeseed, groundnut, sunflower, hazelnut)	The death rate of the *S.* Enteritidis differed with the various oils.	[82]
Brazil	*S*. Enteritidis	Mayonnaise	oil oregano essential oils (OEO1/OEO2) Nisin EDTA	Antimicrobial activity of OEO against *S.* Enteritidis was improved when combined with nisin or EDTA.	[74]
U.K,	*S.* Enteritidis	Mayonnaise	garlic	Garlic (1%) reduced the viable cells of *S.* Enteritidis in mayonnaise by a factor of 10.	[67]
Greece	*S*. Enteritidis	Mayonnais- based Aubergine salad	sorbic/benzoic acids	Addition of preservatives decreased the pathogen survival.	[83]
U.K.	*S*. Enteritidis	Mayonnaise	pH	Within the pH 4.2–4.5 *S*. Enteritidis remained stable for 4 weeks at 4 °C.	[43]
